# Characterisation of *Legionella* Clinical Isolates in Spain from 2012 to 2022

**DOI:** 10.3390/microorganisms12071253

**Published:** 2024-06-21

**Authors:** Juana María González-Rubio, Almudena Cascajero, Beatriz Baladrón, Fernando González-Camacho

**Affiliations:** Legionella Reference Laboratory, National Centre for Microbiology, Instituto de Salud Carlos III, 28220 Madrid, Spain; jmgonzalez@isciii.es (J.M.G.-R.); almudenacd@isciii.es (A.C.);

**Keywords:** *Legionella*, Legionnaires’ disease, clinical isolates, sequence-based typing, outbreaks, public health

## Abstract

Although cases of Legionnaires’ disease are notifiable, data on the phenotypic and genotypic characterisation of clinical isolates are limited. This retrospective study aims to report the results of the characterisation of *Legionella* clinical isolates in Spain from 2012 to 2022. Monoclonal antibodies from the Dresden panel were used for phenotypic identification of *Legionella pneumophila*. Genotypic characterisation and sequence type assignment were performed using the Sequence-Based Typing scheme. Of the 1184 samples, 569 were identified as *Legionella* by culture. Of these, 561 were identified as *L. pneumophila*, of which 521 were serogroup 1. The most common subgroups were Philadelphia (n = 107) and Knoxville (n = 106). The SBT analysis revealed 130 different STs, with the most common genotypes being ST1 (n = 87), ST23 (n = 57), ST20 (n = 30), and ST42 (n = 29). Knoxville has the highest variability with 32 different STs. ST23 is mainly found in Allentown/France (n = 46) and ST42 in Benidorm (n = 18), whereas ST1 is widely distributed. The results demonstrate that clinical isolates show high genetic diversity, although only a few sequence types (STs) are responsible for most cases. However, outbreaks can also occur with rare genotypes. More data on LD and associated epidemiological studies are needed to establish the risk of an isolate causing outbreak in the future.

## 1. Introduction

*Legionella* is a bacterium of environmental origin found worldwide in aquatic environments that can colonise facilities associated with water. It is commonly found in man-made water systems [1]. The bacterium can be inhaled into the lungs through the inhalation of contaminated aerosols.

This bacterium includes more than 70 known species, about half of which have been associated with human disease. *Legionella pneumophila* (*Lpne*) is the main cause of legionellosis, a disease that has two clinical manifestations. Pontiac fever is a non-pnemonic form that is mild and self-limited, with the patient recovering spontaneously. The second clinical manifestation is more serious and is known as Legionnaires’ disease (LD). This is a severe form of pneumonia that has a rapid evolution and can be fatal if adequate treatment is not established. The disease can appear as isolated cases or outbreaks in the community or nosocomial setting. The scale of an outbreak is contingent upon the source of infection. Outbreaks originating from cooling towers have the most significant impact on the population due to the extensive dispersion of aerosols.

*Lpne* is responsible for over 90% of cases of LD [2]. *A* total of 16 different serogroups have been identified for *Lpne*, serogroup 1 (SG1) being the most commonly associated with LD, accounting for approximately 85% of the reported cases [2]. Pneumonia caused by *Legionella* non-*pneumophila* species (*L.* non-*pne*) is rare and mainly affects immunocompromised patients [3], the elderly, or people with comorbidities. Furthermore, with few exceptions, *L*. non-*pne* outbreaks have only been detected in hospital settings [4].

The analysis of LD pathogens by phenotypic and genotypic characterisation of human isolates is valuable for studying the distribution and frequency of *Lpne* strains in epidemiological studies, as well as in outbreak investigations [5,6].

The phenotypic characterisation of the isolates is carried out using the monoclonal antibodies of the Dresden panel [7,8], while the genotypic characterisation is based on the sequence type (ST) [9]. The most common phenotype in clinical isolates is that of the Pontiac group, determined by the presence of the virulence factor encoded by the *Lag-1* gene (monoclonal antibody (MAb) 3/1 positive), whereas the Olda group (MAb 3/1 negative) is the most frequent isolate of environmental sources [10,11]. The phenotype is a polyphyletic characteristic, making it difficult to establish a clear relationship between phenotype/genotype and disease. In recent years, several outbreak studies and regional epidemiological descriptions [12,13] have been published, although up-to-date information at national level is not available.

The Legionella Unit (CNM-ISCIII) is the National Reference Laboratory for legionellosis in Spain. Since the beginning of the 1980s, isolation tests for Legionella have been carried out on clinical and environmental samples, as well as identification and phenotypic and molecular typing tests of isolates of this bacterium. The aim of this study is to present the results of the microbiological diagnosis of LD and the characterisation of clinical isolates by phenotypic and genotypic methods obtained in Spain from 2012 to 2022. The commencement of this period is concurrent with the initiation of the current CNM Microbiological Surveillance Programme. Furthermore, it is also concurrent with the techniques that are currently employed. The data from this study include the characterisation of species, serogroups, subgroups, and sequence types at the national level. In addition, this study analyses the isolates associated with outbreaks and their phylogenetic relationships using a minimum-spanning tree analysis. The results indicate that there is significant genetic diversity in the strains that can cause disease. However, only a few STs account for most cases. Nevertheless, it should be noted that rare STs can also cause outbreaks.

## 2. Materials and Methods

### 2.1. Culture Assay for the Isolation of Legionella from Clinical Samples

A total of 1184 samples for *Legionella* analysis were received at the LRL in Spain between 2012 and 2022. Of these, 819 were respiratory samples (sputum, bronchoalveolar lavage, etc.), 10 were other types of samples such as blood plasma or DNA extracts from respiratory samples, and 355 were clinical *Legionella* isolates obtained in hospitals or microbiology laboratories distributed throughout the country.

To obtain *Legionella* isolates from respiratory specimens a differential culture procedure was performed using Legionella-selective BMPA agar plates. From clinical samples, 100 µL were directly inoculated and spread on a BMPA plate. Another aliquot was treated with Sputasol (OXOID SR0233, Basingstoke, UK) in a 1:1 ratio and heated at 37 °C for 15 min, then subjected to different treatments—direct plating, heat treatment (50 °C for 30 min), and acid treatment (2 M KCl/HCl buffer for 5 min)—before plating. The inoculated plates were incubated at (36 ± 2 °C) for at least 10 days in a humid atmosphere. Suspected *Legionella* isolates were confirmed by differential growth on buffered charcoal yeast extract (BCYE) agar plates with and without L-cysteine.

### 2.2. Identification of Strains

*Legionella* isolates were phenotypically classified as *Lpne* serogrup 1 (SG1), *Lpne* SGs 2–14, or *L.* non-*pne* using a latex agglutination test kit (Legionella latex test kit, Oxoid, UK). Species identification of *L*. non-*pne* isolates was determined by the sequencing of the *mip* gene [14,15].

### 2.3. Sero- and Subgrouping with Monoclonal Antibodies

Both the phenotypic group and subgroup of *Lpne* SG1 and the serogrouping of *Lpne* SG2–SG15 were determined using monoclonal antibodies (MAbs) from the corresponding Dresden panels [7]. The panel employed for the *Lpne* SG1 subgrouping included seven MAbs (Lp1, 3/1, 3, 8/4, 20/1, 26/1, and 10/6). In the case of isolates characterised as SG 2–14, a panel of 16 monoclonal antibodies (MAbs) was employed, with one antibody specific to each serogroup. Antibody binding was detected using rabbit anti-mouse immunoglobulin conjugated to FITC (Dako AS, Glostrup, Denmark). *Lpne* SG1 subgroups were determined according to the flow-chart of Helbiget al. [8].

### 2.4. Genotyping of L. pneumophila by Sequence-Based Typing

Positive *Lpne* isolates were analysed for genotypic identification. Genomic DNA was extracted from bacterial cultures using InstaGene Matrix (Bio-Rad Laboratories, Hercules, CA, USA). Sequence-Based Typing (SBT) was performed according to the ESGLI (version 5.0) [9], based on amplification and sequencing of the seven gene loci—*flaA*, *pilE*, *asd*, *mip*, *mompS*, *proA*, and *neuA*—and/or the *neuAh* allele in some non-SG1 isolates, when the *neuA* allele was not amplified [16]. Sequences reported by Sanger sequencing at the Genomics Core Facility at the ISCIII were submitted to the ESGLI *Legionella* SBT database supported by the UK Health Security Agency, to assign the allelic profile and the ST.

A minimum spanning tree (MST) based on allelic profiles (STs) from *Lpne* culture-positive isolates was constructed and clustering was performed into groups of single-locus variants (SLVs) by using the goeBURST algorithm [17] in PHYLOViZ v2.0 [18].

### 2.5. Multiplex Real-Time qPCR Assay

If an isolate could not be obtained from a respiratory specimen, multiplex real-time polymerase chain reaction was performed. Briefly, DNA was automatically extracted (QiAcube automated system, Qiagen, Hilden, Germany) from clinical samples using the QIAamp DNA Mini Kit. Target genes were the *mip* gene for *Lpne* detection (HEX dye) and the *wzm* gene for *Lpne* SG1 (FAM dye) [19]. *Human ribonuclease P* gene was used as an internal positive control (Cy5 dye) [20]. PCR amplification was performed using SsoAdvanced Universal Probes Supermix (Bio-Rad Laboratories, USA). The assay was performed on the ABI7500 Fast Real-Time PCR System (Applied Biosystems, Foster City, CA, USA).

### 2.6. Nested-PCR Sequence-Based Typing

A nested-PCR Sequence-Based Typing method was performed on negative *Legionella* cultures which had positive qPCR results [21]. Briefly, DNA extracts from clinical samples were used as DNA templates in a first PCR to amplify the seven loci included in the SBT method. The resulting amplification products were then used as DNA templates in a second round of PCR. The amplified PCR products were analysed to determine the allele number. ST were assigned to a complete allelic profile.

### 2.7. Graphical Representation

The data plotted graphically were created using Excel (Windows Microsoft Office 365, 2405 version).

## 3. Results

### 3.1. Identification of Isolates and Phenotypic Characterisation of L. pneumophila

Of 1184 human clinical samples tested, 762 were positive for *Legionella*, 569 were positive by culture, mainly corresponding to *L. pneumophila* (n = 561, 98.5%). A further eight samples were identified as *L.* non-*pne* species, corresponding to *L. bozemanii*, *L. feelei*, *L. longbeachae* (three samples), *L. micdadei* (two samples), and *L. sainthelensis*. *Lpne* DNA was detected in 193 culture-negative respiratory samples. Among the *Lpne* culture-positive isolates, SG1 was the most frequently identified serogroup (91.5%, n = 521). The remaining *Lpne* isolates (n = 40) were classified into SG2–SG14, one of which was a non-typeable serogroup. Of the *Lpne* SG1 isolates (n = 521), 82% belonged to the Pontiac group (n = 428), 9.3% to the Olda group (n = 48), and 1.7% to the Bellingham group (n = 9). Partial analysis with MAbs from the Dresden panel showed “Olda/Bellingham” phenotypic groups (no discrimination between the Olda and Bellingham groups) in 7% of the isolates (n = 36). The subgroup distribution for 350 isolates (81.2%) of the Pontiac group was 25% Philadelphia (n = 107), 25% Knoxville (n = 106), 19% Allentown/France (n = 81), and 13% Benidorm (n = 56); for the remaining 18% no subgroup characterisation was performed. Isolates with the Olda phenotype were mainly subdivided into the OLDA subgroup (n = 43, 89.5%), followed by 6.25% Oxford (n = 3) and 4.2 % Heysham (n = 2).

### 3.2. Temporal Distribution of L. pneumophila SG1 Isolates

**Figure 1:**
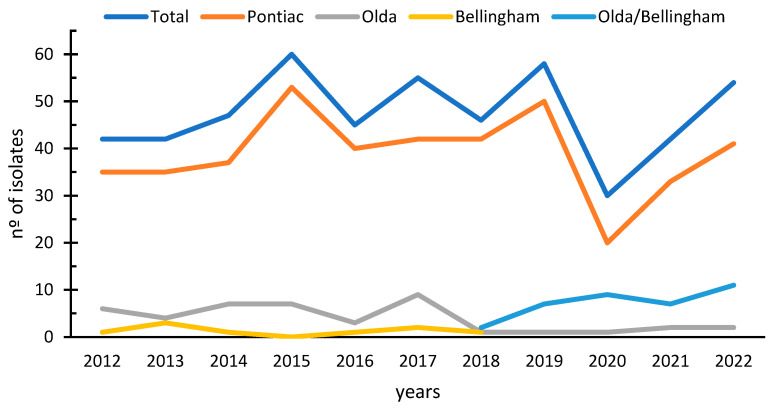
A temporal analysis of the distribution of clinical isolates caused by *L. pneumophila* SG1 throughout the 2012–2022 period.

From 2012 to 2019, the annual average of *Lpne* SG1 isolates was 49. However, in 2020, there was a decrease to 30 isolates. A rebound was observed in 2021 (n = 42), which continued in 2022 with 54 isolates (Figure 1). The graph of the temporal distribution of *Lpne* isolates (Figure 1 light blue line) shows three noticeable peaks. In 2015, the increase in the number of *Lpne* SG1 isolates was correlated with an increase due to the Philadelphia ST899 strain (Pontiac group, Figure 1 orange line), which was the strain isolated from 30 clinical samples from patients related to the Manzanares outbreak (November 2015–February 2016). A slight rebound was also observed in 2017 due to an increase in the Olda group (n = 11) (Figure 1 grey line), which was correlated with sporadic cases of community-associated LD (n = 9) and two nosocomial cases. In 2019, there was a third peak with the appearance of an increase in isolates from the Pontiac group (n = 50), which were distributed in 42 sporadic cases and three nosocomial outbreaks (n = 6 Pontiac Philadelphia and n = 2 Pontiac Benidorm).

The number of cases decreased by 40% in 2020 (n = 30) compared to the previous year, which coincided with the COVID-19 pandemic and anti-COVID measures. However, there was a rebound in 2021 (n = 42), which continued in 2022 with 54 isolates surpassing pre-pandemic media.

Since 2018, the full panel of Dresden monoclonal antibodies has no longer been utilised. As a result, it is no longer possible to distinguish between Olda/Bellingham groups or discriminate between subgroups (Figure 1 dark blue), except for outbreak-associated isolates where the full panel is still employed.

### 3.3. Analysis and Genotypic Characterisation of Legionella pneumophila

The SBT analysis identified 134 different STs, with 57.6% of the isolates (n = 323) belonging to 11 different STs. The most frequent ST was ST1 (n = 87), followed by ST23 (n = 57), ST20 (n = 33), ST899 (n = 30), ST42 (n = 29), ST181 (n = 24), ST37 (n = 20), ST448 (n = 13), ST48 (n = 12), ST62 (n = 10), and ST94 (n = 9). However, 17.8% of the clinical strains (100 cases) corresponded to isolates with a unique ST (n = 1).

**Figure 2:**
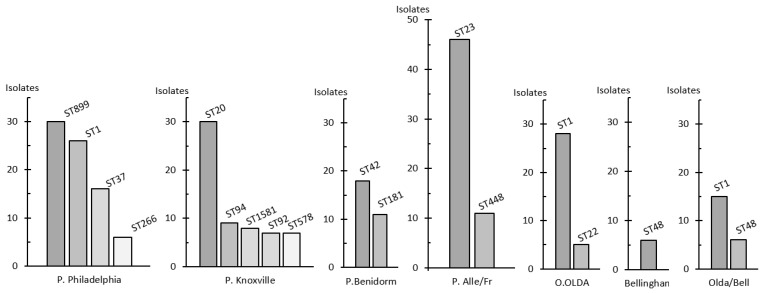
Distribution of the most frequent isolates by phenotype and sequence type.

The subgroups of *Lpne* SG1 showed the greatest diversity of ST. The Knoxville subgroup had 32 different STs, and the Philadelphia subgroup had 21 different STs. ST1 was the most widely distributed, mainly by the Philadelphia (n = 26) and OLDA (n = 28) subgroups; in addition, the three isolates from the Oxford subgroup were ST1. Furthermore, ST23 and ST42 were only characterised in the Pontiac group. ST23 was found in 88.5% (n = 46) of isolates belonging to the Allentown/France subgroup, while ST42 was present in 90% of cases in Pontiac Benidorm. ST899 was exclusively found in Philadelphia isolates from the Manzanares outbreak. Finally, ST20 was predominantly found in the Knoxville subgroup (91%, n = 30), although it was also present in the Heysham and Allentown/France subgroups as well as the Olda/Bellingham group (n = 1 each) (Figure 2).

The non-SG1 isolates exhibited a wide diversity of STs, with 28 STs identified. None of these STs matched those found in SG1, and there was no overlap between the STs of the SG2–SG10 isolates, except for ST93 which was present in SG3 and SG6, and ST1326 which was described in both serogroup SG4 and SG10 (Table 1).

**Figure 3:**
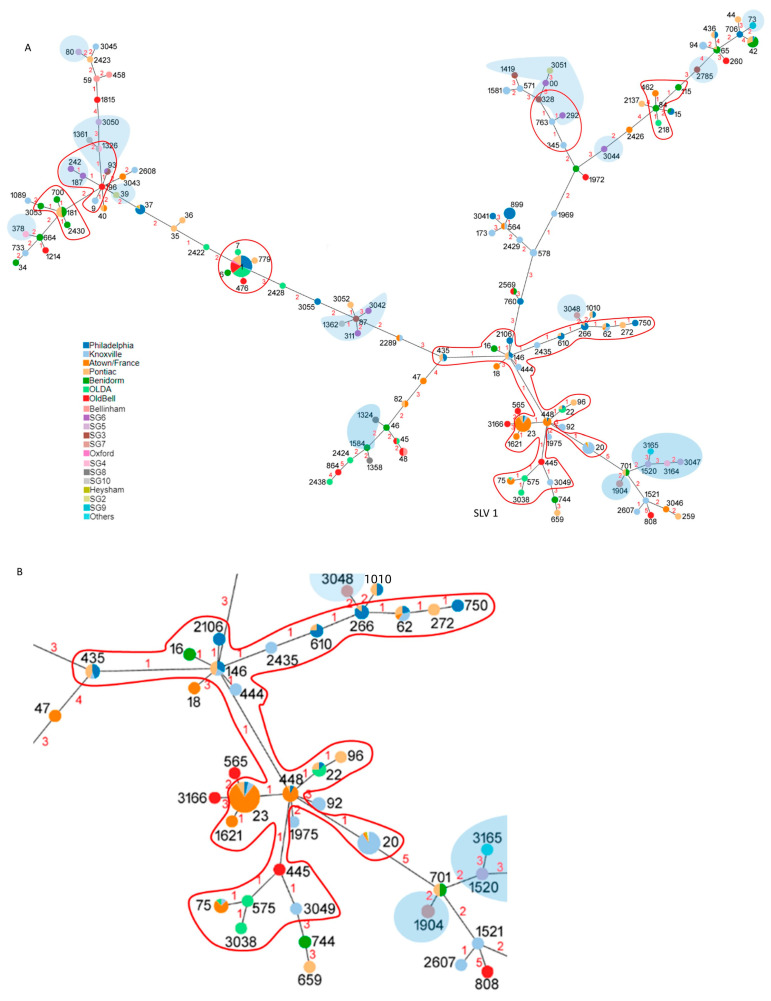
(**A**) Minimum-spanning tree analysis of 561 isolates obtained with PHYLOViZ for the ST allelic profiles. The pie charts represent the STs, with the size of each circle proportional to the number of isolates. The different colours of the pie charts refer to the phenotypic characteristics. The ST numbers are shown in black. The numbers on the branches represent the number of difference loci (in red). SLVs are indicated by red outlines, and non-SG1 are indicated by shaded backgrounds. (**B**) Enlarged image of SLV 1.

A phylogenetic tree was constructed using the ST allelic profiles of *Lpne* culture-positive isolates. Six clusters of single-locus variants (SLVs) were identified, each comprising at least four different STs (Figure 3). The largest cluster (Figure 3B) was formed by 173 isolates (31%) belonging to 22 different STs. Conversely, non-SG1 isolates are dispersed throughout the phylogenetic tree.

### 3.4. Distribution of Phenotypes and Genotypes of Strains More Frequently Identified in Sporadic Cases and Outbreaks

**Figure 4:**
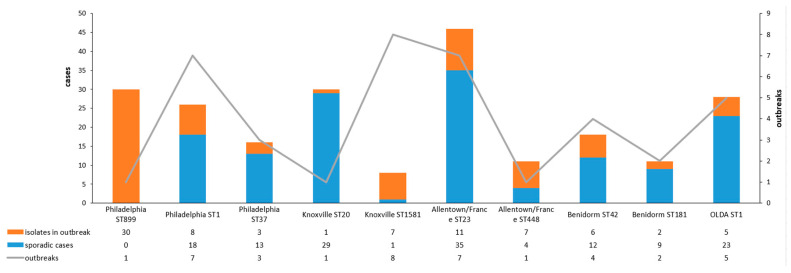
Distribution of isolates with phenotype and sequence type more frequently described in the study and their relationship with the number of isolates obtained in sporadic cases and outbreaks.

An analysis of the relationships between the phenotypic and genotypic isolates and their distribution in sporadic cases and in outbreaks is shown in Figure 4.

The strain Allentown/France ST23 is the most frequently isolated from clinical cases (n = 46) and appears to be very widely distributed among both sporadic cases (n = 35) and 11 cases associated with outbreaks (seven outbreaks). One outbreak associated with a hotel yielded five clinical specimens from a total of 44 patients [22].

Knoxville ST20 is the second strain with the highest number of clinical cases (n = 30), mainly associated with sporadic cases of LD, except for one case associated with an outbreak of LD in a residential home for the elderly. This strain appears to be widely distributed throughout Spain.

All the isolates presenting ST899 corresponded to the Philadelphia subgroup from patients affected by an outbreak in Manzanares in 2015 [23]. Additionally, ST899 was detected by nested-PCR SBT in 37 clinical samples, also related to the same outbreak, despite negative culture results. Of the other Philadelphia isolates, 26 were assigned to SG1, n = 18 to sporadic cases, and eight cases to different outbreaks. In addition, 16 isolates were identified with ST37, three of which were linked to distinct outbreaks (one isolate per outbreak).

The Benidorm subgroup was predominantly represented by the ST42 genotype in 18 isolates, six of which were associated with four outbreaks reported during the studied period, and ST181 was found in 11 isolates, two of which were associated with two independent outbreaks.

Allentown/France ST448 was found in seven isolates from clinical samples in the same outbreak [24] and was also described in four sporadic cases.

Finally, Knoxville ST1581 was responsible for an outbreak in 2014 (Sabadell-Ripollet) involving seven clinical samples; in addition, a sporadic case was linked to this isolate.

OLDA ST1 has been characterised in 28 isolates, mostly associated with sporadic cases, but in five different outbreaks causing five clinical patients.

## 4. Discussion

The data collected from the isolates with positive cultures showed a higher percentage of *Lpne* SG1 (91.5%) than those reported in the last ECDC surveillance report (82%) [2]. In contrast, the percentage of *L.* non-*pne* was lower (1.5%) compared to the previous report (3%). This discrepancy may be attributed the prior characterisation conducted by hospitals prior to sample submission, with greater attention devoted to samples from patients with a positive UAT result.

More than 70 species of *Legionella* have been identified, with 24 of these capable of causing infection. *Lpne* is the most prevalent pathogenic species, although in New Zealand and Australia, LD caused by *L. longbeachae* is as common as *Lpne* [25]. Conversely, LD caused by the other *Legionella* non-*pne* are very rare and predominantly observed in immunocompromised patients and elderly people [3,26].

With regard to global published data on phenotypic characterisation, these are scarce. This may be due to the limited availability of antibodies for immunofluorescence typing and the limited number of clinical isolates available. Additionally, the diagnosis is predominantly made by UAT, with biological samples only taken on rare occasions. The data available in our laboratory indicate that the Pontiac group (MAb 3/1 positive) is the most common phenotype, with an incidence of 82% in clinical isolates. These results are in line with previously published data [10].

In terms of the temporal distribution of LD cases, after the decrease during the COVID-19 pandemic, an increase has been observed in the last two years. Specifically, the number of cases in 2022 was higher than the average value of the 8 years before the pandemic. This trend is consistent with data published by the ECDC [2], which also show an increasing trend in LD cases. This temporal distribution shows the same trend line as the legionellosis notification rate in Spain for the same period [27]. It shows a peak in 2015, a decrease in 2020, and an upward trend for 2021 and 2022.

The genotypic characterisation revealed a high degree of variability, with up to 130 different STs identified. However, only 11 STs accounted for 57.6% of the total isolates. This high level of genetic diversity has also been demonstrated in other studies [10,28]. The most commonly defined sequence types in this study were ST1, ST23, ST20, ST899, and ST42. ST1 and ST20 exhibited a global distribution among phenotypes, whereas ST23 and ST42 were predominantly found in the Pontiac group. ST23 was associated with the Allentown/France subgroup, and ST42 was described in the Benidorm subgroup. These results are comparable to those of a retrospective study conducted for Slovenia between 2006 and 2020. In this study, the most prevalent strains were identified as ST1 and ST23. ST1 is predominantly associated with the Philadelphia subgroup, whereas ST23 is primarily linked to the Allentown/France subgroup [5].

The non-SG1 isolates exhibited a high genotypic diversity, with none of these coinciding with the STs found in SG1. Furthermore, the distribution of all non-SG1 in the phylogenetic tree constructed from the allelic profiles of the sequence type shows a dispersion throughout all the branches, which indicates their polyphyletic character. Conversely, the clusters identified by SLV group indicate a low number of STs that contain a low number of isolates, except for the cluster that contains ST1 (five different STs, n = 91) and the largest cluster, which contains ST23 and ST20 (22 different STs, n = 173). This cluster encompasses 31% of the clinical isolates, which could be related to greater pathogenicity. Given the limited information provided by STs, further investigation is required to gain a deeper understanding of this phenomenon. Genomic studies involving higher resolution are necessary to identify genetic markers of pathogenicity.

The occurrence of common STs in relation to the distribution of cases identified as either sporadic or as outbreaks also showed variability. ST1 and ST20 were mainly found in sporadic unrelated cases, whereas ST23 was associated with both outbreaks and sporadic cases. This distribution of related cases with ST23 has previously been described in Italy [29]. Previous studies have examined clinical *L. pneumophila* isolates at a regional level in Spain, specifically in the Valencian Community (VC) [12] and in Catalonia [13]. In the VC study, the most frequent STs were ST1, ST578 (endemic to a local region [30]), and ST23. However, the phenotypic characterisation of these isolates was not performed. In Catalonia, analysis of clinical isolates revealed that ST37, ST23, and ST1 were the most frequent, with Philadelphia ST37 and Philadelphia ST32 being the most prevalent strains.

Although there are predominant phenotypes–genotypes associated with clinical cases, the data described in this work suggest that a wide spectrum of phenotypes and genotypes could appear. There may be rare or previously unseen cases associated with ST that could lead to outbreaks, as was the case with ST899 in Manzanares, Spain.

This study presents an LD analysis of the samples received throughout the country over the past 11 years. However, the main limitation of the study is that clinical samples for detection of *Legionella* can be sent to other regional laboratories. Furthermore, there appears to be a bias towards SG1 in terms of the selected samples that hospitals send to our laboratory.

Phenotypic characterisation data indicate that the virulence factor encoded by the *lag-1* gene, which is detected by MAb 3/1 (Pontiac group), is present in most clinical isolates. In contrast, in studies of environmental isolates, the majority factor is the Olda group [10,11], which in clinical samples appears to be associated with immunocompromised patients [31]. Conversely, with regard to genotypic characterisation by ST, our data indicate the existence of a principal cluster of STs related to clinical cases, with only a few STs observed in the majority of cases. However, there is no discernible correlation between virulence and ST. Consequently, in the Prevention and Legionellosis Surveillance Programmes, to assess the risk posed to *Legionella,* the phenotypic characterisation of the isolates remains a valuable tool. Conversely, the ST provides valuable information for epidemiological and structural studies of *Legionella* populations, although in some cases it is insufficient. The limited availability of antibodies and the low resolution of the ST favour the use of whole-genome sequencing (WGS) [32,33,34] or core genome multilocus sequence typing (cgMLST) [35,36] techniques in outbreak investigations. This trend necessitates the establishment of consensus and standardisation of these tools for *Legionella*.

## Figures and Tables

**Table 1:** Sequence type of *Legionella pneumophila* SG2–SG10.

Serogroup 2–10	Number of Isolates	ST
2	2	ST39, ST3051
3	6	ST87, ST93 (x2), ST328, ST1419, ST2785
4	3	ST378, ST1326, ST3164
5	7	ST80 (x2), ST1520 (x2), ST3047 (x2), ST3050
6	8	ST93, ST187, ST242, ST292, ST311, ST3042, ST3044, incomplete ST
7	6	ST1904 (x5), ST3048
8	3	ST1324 (x2), ST1358
9	1	ST73
10	3	ST1326, ST1361, ST1362
non-typeable	1	ST3165

## Data Availability

The original contributions presented in the study are included in the article, further inquiries can be directed to the corresponding author.
